# Epigenomic analysis of *KLF1* haploinsufficiency in primary human erythroblasts

**DOI:** 10.1038/s41598-021-04126-6

**Published:** 2022-01-10

**Authors:** Steven Heshusius, Laura Grech, Nynke Gillemans, Rutger W. W. Brouwer, Xander T. den Dekker, Wilfred F. J. van IJcken, Benjamin Nota, Alex E. Felice, Thamar B. van Dijk, Marieke von Lindern, Joseph Borg, Emile van den Akker, Sjaak Philipsen

**Affiliations:** 1 Department of Hematopoiesis, Department of Blood Cell Research, Sanquin Research, Plesmanlaan 125, 1066 CX Amsterdam, The Netherlands; 2 Department of Cell Biology, Erasmus MC, P.O. box 2040, 3000 CA Rotterdam, The Netherlands; 3 Department of Applied Biomedical Science, Faculty of Health Sciences, Mater Dei Hospital, Msida, MSD2090 Malta; 4 Center for Biomics, Erasmus MC, P.O. box 2040, 3000 CA Rotterdam, The Netherlands; 5 Department of Molecular and Cellular Hemostasis, Sanquin Research, Plesmanlaan 125, 1066 CX Amsterdam, The Netherlands; 6 Thalassaemia Testing and Haemoglobin Research Laboratory, University of Malta, and Thalassaemia Clinic, Mater Dei Hospital, Msida, MSD2090 Malta

**Keywords:** Cell biology, Genetics, Molecular biology

## Abstract

Haploinsufficiency for the erythroid-specific transcription factor KLF1 is associated with hereditary persistence of fetal hemoglobin (HPFH). Increased HbF ameliorates the symptoms of β-hemoglobinopathies and downregulation of KLF1 activity has been proposed as a potential therapeutic strategy. However, the feasibility of this approach has been challenged by the observation that *KLF1* haploinsufficient individuals with the same *KLF1* variant, within the same family, display a wide range of HbF levels. This phenotypic variability is not readily explained by co-inheritance of known HbF-modulating variants in the *HBB*, *HBS1L-MYB* and/or *BCL11A* loci. We studied cultured erythroid progenitors obtained from Maltese individuals in which *KLF1* p.K288X carriers display HbF levels ranging between 1.3 and 12.3% of total Hb. Using a combination of gene expression analysis, chromatin accessibility assays and promoter activity tests we find that variation in expression of the wildtype *KLF1* allele may explain a significant part of the variability in HbF levels observed in *KLF1* haploinsufficiency. Our results have general bearing on the variable penetrance of haploinsufficiency phenotypes and on conflicting interpretations of pathogenicity of variants in other transcriptional regulators such as *EP300*, *GATA2* and *RUNX1*.

## Introduction

β-hemoglobinopathies such as β-thalassemia and sickle cell anemia (SCA) are caused by mutations within the β-globin subunit of adult hemoglobin (HbA, α2β2). High levels of fetal hemoglobin (HbF, α2γ2) ameliorate the symptoms of β-thalassemia and SCA. Reactivation of fetal γ-globin expression and, in the case of SCA, concomitant downregulation of sickle β-globin expression is therefore seen as an attractive approach to improve the condition of β-hemoglobinopathy patients. Although not completely resolved, fetal-to-adult hemoglobin switching depends on a core network of transcriptional regulators converging on B-cell lymphoma 11A (BCL11A) and Lymphoma Related Factor (LRF, encoded by *ZBTB7A*), that are direct repressors of the *HBG1/2* genes encoding γ-globin^1–3^. In erythroid cells, *BCL11A* and *ZBTB7A* expression is induced by the transcription factor Krüppel-like factor 1 (KLF1)^4–6^. Haploinsufficiency for *KLF1* results in hereditary persistence of fetal hemoglobin (HPFH) through a mechanism that involves reduced expression of BCL11A^4, 6^ and LRF^5^. Modulation of this regulatory network has been proposed as an approach to increase HbF levels^1–10^, a notion that has been confirmed in animal models^11–15^. As BCL11A^16^ and LRF^17^ are tumor suppressors with important functions outside erythropoiesis, targeting the correct cells is important. In addition, KLF1^18, 19^ and LRF^20^ are essential for terminal erythroid differentiation. *KLF1* variants are associated with a broad spectrum of benign but also severe erythroid defects^9^. These include the In(Lu) and Indian blood types^21^, pyruvate kinase deficiency^22^, increased HbA2 (α2δ2)^23^, increased zinc protoporphyrin^24^, congenital dyserythropoiesis^25–28^ and HPFH^4^. In families with HPFH caused by *KLF1* haploinsufficiency, HbF levels are variable suggesting additional regulators^4^. This variation could only be partially explained by coinheritance of known HbF-modulating genotypes at the *HBB* locus itself and *trans*-acting HPFH loci *BCL11A* and *HBS1L-MYBI*^4, 7, 9^. This raises the possibility that another, as yet unidentified, *trans*-acting locus is involved. To investigate the phenotypic variability of *KLF1* haploinsufficiency, we used cultured erythroid cells carrying the *KLF1* p.K288X variant obtained from two Maltese HPFH pedigrees^4^ and control Maltese individuals. This nonsense variant, now annotated as SNP rs267607202, ablates the complete zinc finger domain and therefore abrogates DNA binding of the mutant protein. Since the truncated KLF1 protein failed to rescue the HPFH phenotype, the *KLF1* p.K288X variant is considered to be a *null* mutation^4, 9^. Here, we used gene expression (RNA-seq) and chromatin accessibility assays (ATAC-seq^29^) to demonstrate that *KLF1* haploinsufficiency affects erythroid gene expression of KLF1 target genes, but -with the exception of a small set of genes including *HBG1/2*- chromatin accessibility was not altered. The HbF expression in HPFH samples correlated negatively with *KLF1*, *BCL11A* and *ZBTB7A* expression levels. We suggest that allelic variation affecting wildtype *KLF1* expression explains a significant part of the HbF variability observed within this cohort of Maltese HPFH individuals.

## Results

### *KLF1* haploinsufficiency marginally affects in vitro erythropoiesis but induces HPFH

**Figure 1:**
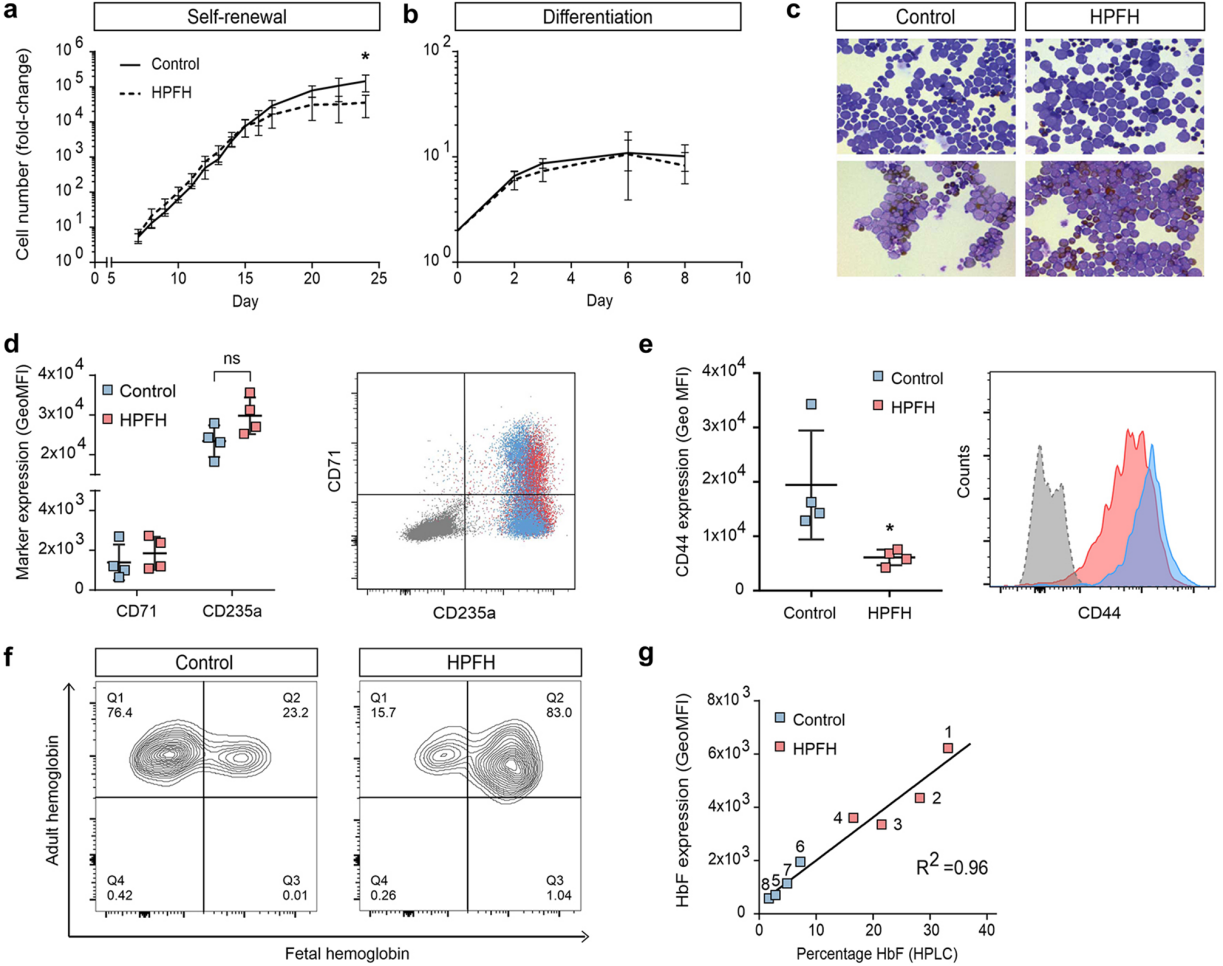
Cultured erythroblasts from *KLF1* p.K288X carriers reflect the HPFH phenotype (**a**) Cell counts from expansion stage of erythroid cultures started from PBMCs. Final cell count after 24 days 1.4 × 10^11^ ± 7.3 × 10^10^ (controls) vs. 3.6 × 10^10^ ± 2.2 × 10^10^ (HPFH), mean ± sd, N = 3–4, * = *P* < 0.05 Students T-test. (**b**) Cell counts from erythroid differentiation stage of culture. (**c**) Cell morphology at two sampling time points. Top: erythroblast after 10 days of expansion from PBMCs. Bottom: erythroblasts after 48 h of differentiation. (**d**) Surface expression of differentiation markers transferrin receptor (CD71) and glycophorin A (CD235a). Left: quantification of Geometric Mean Fluorescent Intensity (GeoMFI) after correction for isotype control. Right: representative dot plot for control sample in blue and HPFH in red, isotype in grey. mean ± SD, N = 4; ns = p > 0.05 Students T-test. (**e**) Surface expression for CD44 (Indian blood group). Left: quantification of GeoMFI after correction for isotype control. Right: representative histograms for control sample in blue and HPFH in red, isotype in grey. Mean ± SD, N = 4; * = *P* < 0.05 Students T-test. (**f**) Flow cytometry after intracellular staining for HbA and HbF. Two representative contour plots are shown. (**g**) Correlation between HbF levels determined by HPLC and flow cytometry; the individuals from whom the samples were derived are indicated.

Maltese *KLF1* p.K288X carriers, further denoted as HPFH individuals, provide an opportunity to study the effect of reduced levels of this crucial transcriptional regulator on adult human erythropoiesis^4^. To study the effect of *KLF1* haploinsufficiency on the transcriptome and chromatin accessibility of human erythroid cells, we isolated peripheral blood mononuclear cells from four Maltese HPFH individuals, and four Maltese and two Dutch control donors (Table 1, Suppl Table 1, Suppl Fig. 1) and differentiated these towards the erythroid lineage^30–32^. Initially, HPFH and control cultures showed similar expansion potential. However, during late expansion (> 15 days) control cultures expanded two- to three-fold more compared to HPFH cultures (Fig. 1a). Terminal differentiation induced a short proliferation phase (3 days) followed by growth arrest (Fig. 1b) and morphological changes, which were comparable between HPFH and control cells (Fig. 1c). Based on CD71 (transferrin receptor) and CD235a (glycophorin A) expression and overall hemoglobinization, no major differences in erythroid differentiation were observed (Fig. 1d; Suppl Fig. 2). The previously identified KLF1 target gene and cell surface marker CD44 was expressed at lower levels on cells from HPFH cultures (Fig. 1e)^4, 21^. Flow cytometry for HbA and HbF at 96 h of differentiation showed a population expressing only HbA and a population expressing both HbF and HbA (Fig. 1f). HbF levels were higher in HPFH compared to control cultures and generally recapitulated the relative expression levels of HbA and HbF in vivo (Fig. 1f; Table 1, Suppl Table 1). The geometric mean fluorescence intensity (GeoMFI) correlated to the percentage of HbF measured by HPLC on both cultured erythroid cells (Fig. 1g) and erythrocytes (Suppl Fig. 2b), but no significant increase in HbF GeoMFI within the HbF/HbA double-positive cells was observed (Suppl Fig. 2c). This shows that higher HbF results from more cells expressing HbF as opposed to increased HbF expression per cell (Fig. 1f). As reported before, control and HPFH cultures showed higher HbF levels compared to erythrocytes obtained from the same individuals (Fig. 1f; Suppl Fig. 2b; Table 1)^4^. For three out of four HPFH individuals, the HbF levels ranked identical for erythrocytes and cultured erythroid cells (Table 1). For HPFH individual 2, HbF ranked low in erythrocytes (1.3% HbF) but second highest in cultured cells (28.1% HbF; Table 1). Total hemoglobinization was comparable between control and HPFH samples (Suppl Fig. 2a), indicating that increased HbF involved specific up-regulation of *HBG1/2* expression and was not the consequence of overall lower HbA levels. Collectively, the reduced CD44 expression along with the observed ranking in HbF levels show that the erythroid cultures recapitulate the *KLF1* haploinsufficiency phenotypes^4, 7, 9, 21^.

**Table 1:** The study.

Sample No	Individual denominator	*KLF1* genotype	HbF In vivo	HbF Culture	HbA1 Culture	ATAC-seq	RNA-seq
1	FamF, m2	wt/p.K288X	*12.32*	33.07	46.41	Yes	Yes
2	FamD, m8	wt/p.K288X	*1.34*	28.13	54.15	Yes	Yes
3	FamF, m5	wt/p.K288X	*7.26*	21.52	61.43	Yes	Yes
4	FamF, m4	wt/p.K288X	*3.8*	16.59	62.36	Yes	Yes
5	FamF, m1	wt/wt	*0.16*	2.53	85.55	No	Yes
6	FamF, m3	wt/wt	*0.6*	7.22	77.02	Yes	Yes
7	FamD, m7	wt/wt	*0.17*	5.02	82.77	Yes	Yes
8	unrelated, m9	wt/wt	*0.3*	2.18	86.44	Yes	Yes
9	unrelated, d1	wt/wt	< *0.5*	n.d	n.d	No	Yes
10	unrelated, d2	wt/wt	< *0.5*	n.d	n.d	No	Yes

Individual denominators: see Suppl Fig. 1 for pedigree trees of FamD and FamF, and Suppl Table 1 for additional data on FamD and FamF family members. ‘m9’ indicates unrelated Maltese individual; ‘d1’ and ‘d2’ indicate unrelated Dutch individuals. See Borg et al.^4^ for description of FamF.

### *KLF1* haploinsufficiency results in deregulated gene expression

**Figure 2:**
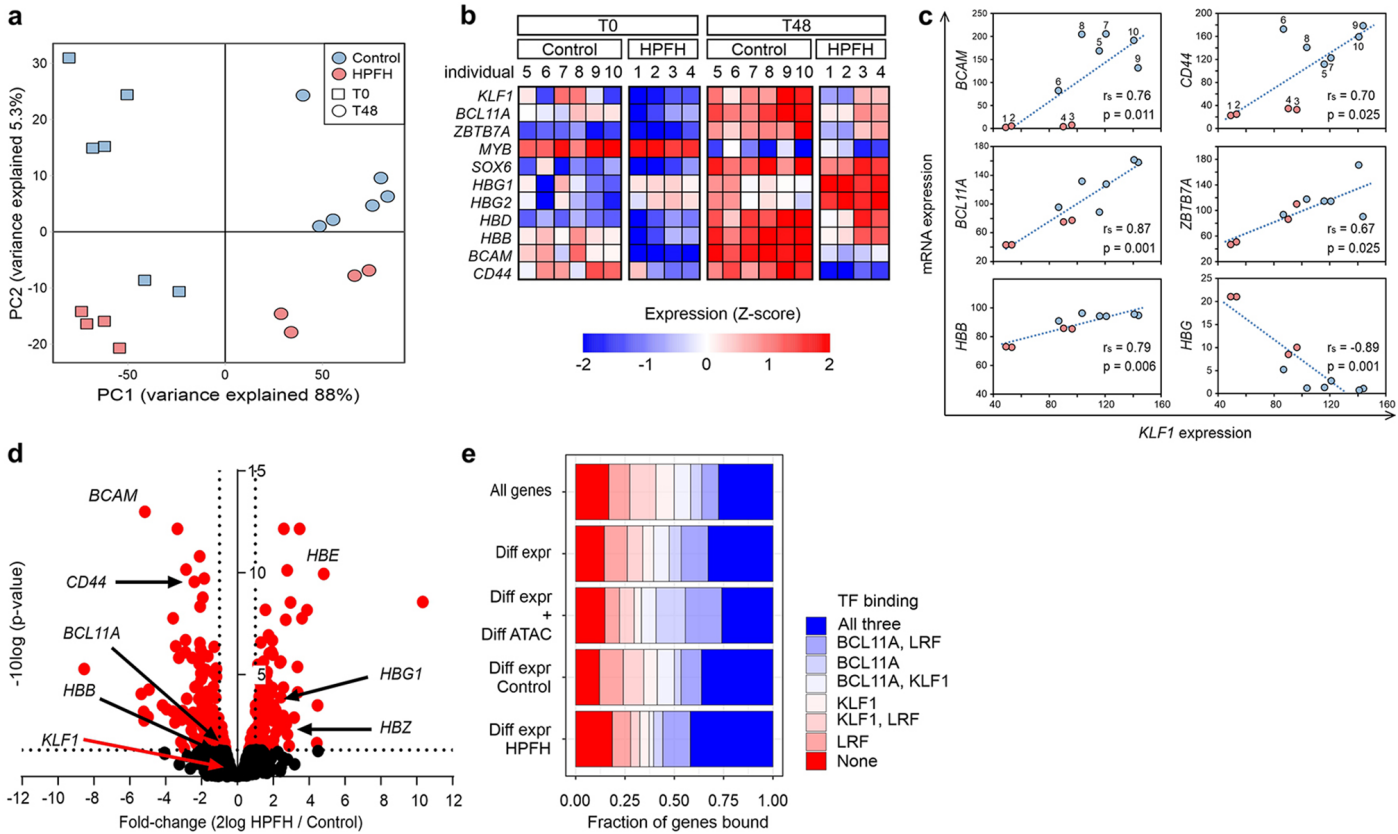
RNA-seq analysis of cultured erythroblasts. RNA-seq was performed on six controls and four HPFH individuals at the start of differentiation (T0) and after 48 h of differentiation (T48). (**a**) Principal component (PC) analysis for 2000 most variable genes (blue: controls; red: HPFH individuals; squares: T0 samples, circles: T48 samples). (**b**) Heatmap for selected transcription factors and globins from *HBB* locus at T0 and T48; the individuals from whom the samples were derived are indicated (Table 1). Scale bar indicates Z-transformed 2log expression values (CPM). (**c**) mRNA expression for selected globins and regulators plotted against KLF1 expression at T48; the individuals from whom the samples were derived are indicated (Table 1). (**d**) Volcano plot with differentially expressed genes highlighted in red at T48 (FDR < 0.05). (**e**) Integration with ChIP datasets for KLF1, BCL11A and ZBTB7A. Bar plot shows fraction of genes with ChIP signal in gene sets from differential expression analysis on RNA-seq and ATAC-seq datasets at T48. ρ: Spearman's rank correlation coefficient. CPM: counts per million mapped reads.

Next, we asked which other genes were affected by *KLF1* haploinsufficiency. We isolated RNA from HPFH and control samples grown under expansion conditions (T0) and after 48 h of differentiation induction (T48, Fig. 1c, Suppl Fig. 2d). RNA samples were poly-A enriched prior to mRNA sequencing. Principal component analysis (PCA) separated the two sampling time points (first component) and HPFH from controls (second component, Fig. 2a; more pronounced during differentiation). This was also reflected in the correlation matrix (ρ < 0.93 and ρ > 0.95, Suppl Fig. 3a). At T48, out of 9136 unique RNAs we identified 344 differentially expressed genes (3.7%; FDR < 0.05) between HPFH and control samples with 173 up- and 171 downregulated genes (Suppl Table 2a,b). In line with increased HbF expression, HPFH erythroblasts expressed higher γ-globin mRNA levels (*HBG1/2)* and lower β-globin mRNA levels (*HBB)* compared to controls (Fig. 2b,c). Expression of known KLF1 target genes *BCAM* and *CD44*^4, 21^ was reduced in HPFH samples (Fig. 2d, Suppl Table 2a).

Of a set of known globin regulators only *BCL11A* was significantly lower expressed (1.8-fold) in HPFH compared to control cultures (FDR < 0.05; Fig. 2b, Suppl Fig. 3b)^4^. Of note, *KLF1* and *ZBTB7A,* the gene encoding LRF, were expressed approximately 1.6-fold lower, but these genes did not pass the correction threshold for multiple testing (*P*-value < 0.001, FDR > 0.05). RT-qPCR validated the different expression levels of *BCL11A, HBG1/2*, and *HBB*, but expression of *KLF1* and *ZBTB7A* genes was not significantly different comparing control to HPFH samples (Suppl Fig. 3c). We previously reported that the KLF1 p.K288X mRNA may be subject to nonsense mediated decay and that KLF1 mRNA expression was lower in samples from HPFH individuals^4^. The stringent threshold applied here combined with heterogeneity between the samples may explain the inability to detect significant expression differences for *KLF1* and *ZBTB7A.* Within individual samples, lower *KLF1* expression correlated with lower expression of *BCL11A* and *ZBTB7A*, and higher expression of *HBG1/2*. (ρ = 0.90 *P* < 0.001, ρ = 0.67 *P* < 0.05, ρ = − 0.71 *P* < 0.05 respectively, Fig. 2c). In addition, *HBG1/2* expression levels correlated well with the percentage of HbF found in the cultures (see also Fig. 1g). This suggests that the observed variability in HbF levels results from additional variation of *KLF1* expression in the haploinsufficiency setting of the HPFH samples. Many genes change expression at the onset of differentiation^33^. Indeed, out of the 344 differentially expressed genes between HPFH and control erythroblasts, 296 were also found to change upon differentiation induction (T0 and T48; FDR < 0.05; Suppl Fig. 3c). Importantly, differential expression between HPFH and control samples was not due to an altered response to induction of differentiation (Fig. 1c,d). To check for association with KLF1 DNA binding*,* the differentially expressed genes were referenced against a ChIP-seq dataset for KLF1 in HUDEP2 cells^5^. Of the 344 deregulated genes, 49% displayed a KLF1 peak (Fig. 2e; Suppl Table 2b,c), most of which were located in the promoter or first intron (83%; Suppl Fig. 3d). This was not enriched when compared to KLF1 peaks identified in the expressed genes of the total RNA-seq dataset (58% of genes; Fig. 2e)*.* Next, datasets from BCL11A and LRF ChIP-seq experiments^2^ were included to check for indirect effects of *KLF1* haploinsufficiency. Genes bound by KLF1, BCL11A and/or LRF represented 81% (278/344) of differentially expressed genes (Fig. 2e, Suppl Table 2b,c). Interestingly, 31% (108/344) of the differentially expressed genes were bound by BCL11A and/or LRF, but not by KLF1 (Suppl Table 2b,c), supporting the notion that *KLF1* haploinsufficiency partly acts through BCL11A and LRF^4, 5^. Collectively, this suggests that the 344 differentially expressed genes result from an interplay of regulation by KLF1, BCL11A and LRF. To assess whether this is reflected in alterations in chromatin accessibility, Transposase Accessible Chromatin (ATAC) sequencing was used^29^.

### Gene-proximal chromatin accessibility is largely unchanged in *KLF1* haploinsufficiency

**Figure 3:**
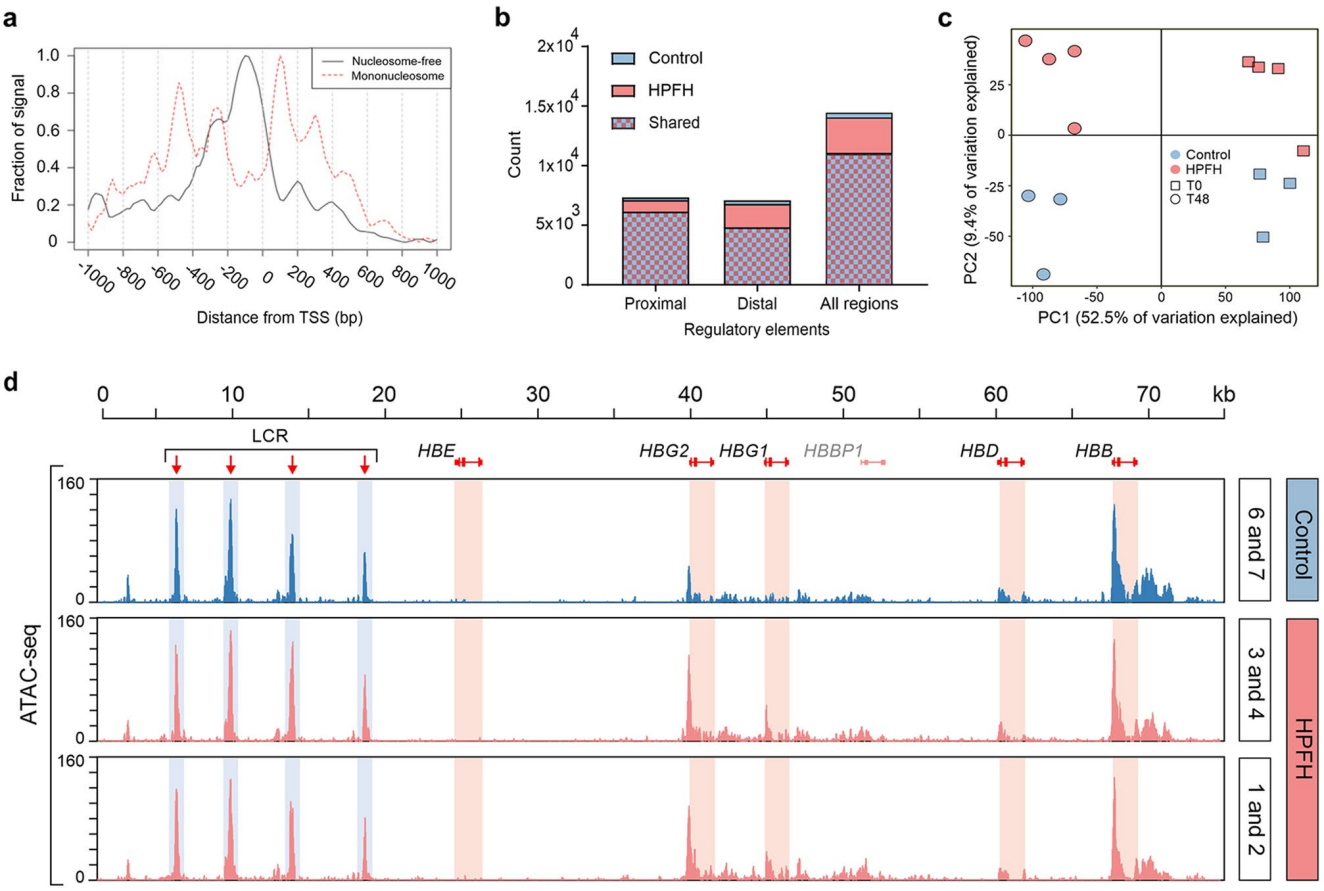
ATAC-seq analysis of cultured erythroblasts. ATAC-seq was performed on three controls and four HPFH individuals at the start of differentiation (T0) and after 48 h of differentiation (T48). (**a**) Location of ATAC peaks relative to the transcription start site (TSS), at T48 (**b**) Quantification of regulatory regions, determined by MACS2 and ALTRE, at T48 (**c**) Principal component (PC) analysis on all ATAC peaks that change between T0 and T48 (blue: controls; red: HPFH individuals; circles: T0; squares: T48) (**d**) Genomic tracks showing ATAC signals across the *HBB* locus for combined control samples (individuals 6 and 7, 7% and 5% HbF (Table 1)) and HPFH samples (individuals 3 and 4, 22% and 17% HbF; individuals 1 and 2, 33% and 28% HbF (Table 1)), at T48. Data range (raw counts) is indicated. LCR: locus control region.

To address if *KLF1* haploinsufficiency controls specific chromatin accessibility, for instance on the deregulated target genes, the HPFH and control cells were subjected to ATAC sequencing. Fragment length in ATAC-seq showed the expected periodicity^29^, with more insert sizes at multiples of around 200 bp, corresponding to the lack of one or more nucleosomes (Suppl Fig. 4a, insert). Peaks were defined as present in HPFH or control samples when detected in three or more replicates of that group. ATAC-seq peaks around 200 bp in length were enriched just before the transcription start sites (TSS; Fig. 3a). To detect differences in the abundance of specific regulatory elements, open regions were categorized as belonging to proximal or distal regulatory elements based on distance to the nearest TSS (ALTRE R-package)^34^. 14,654 putative regulatory elements were identified, with 76% found in both HPFH and control samples. 4% of all ATAC peaks were unique to controls and 20% to HPFH samples (Fig. 3b). Of these HPFH-specific elements the majority were identified at distal locations (> 5000 bp from the TSS). Next, the reads per called peak were quantified and annotated to the nearest TSS within a window of + 5000 bp upstream and − 3000 bp downstream, yielding 41,447 peaks. To determine the degree of variance in chromatin accessibility we performed PCA on these peaks. Samples from the two timepoints cluster along the first component, accounting for ~ 52% of total observed variance (Fig. 3c). This most likely reflects the changes in accessible chromatin regions during differentiation^35^. In the second component, the HPFH samples clustered separately from the controls (Fig. 3c). However, this separation was lost upon restricting the analysis to the 2000 most variable ATAC-seq peaks (Suppl Fig. 4c). This indicates that *KLF1* haploinsufficiency only accounts for minor variations in chromatin accessibility in the 2000 most variable regions. At T48, 559 out of 41,447 peaks showed altered accessibility in HPFH samples compared to controls (EdgeR^36^; FDR < 0.05, Suppl Table 2d); 339 were more and 220 were less accessible (Suppl Fig. 4c). For comparison, the variation in chromatin accessibility observed during erythroid differentiation identified 9338 differential peaks between T0 and T48. The minor differences in chromatin accessibility between HPFH and control samples against the larger scale rearrangements that occurred during differentiation illustrate the limited effect of *KLF1* haploinsufficiency on chromatin accessibility in erythroid cells. A notable exception was within the *HBB* locus with HPFH samples showing increased accessibility of the *HBG1* and *HBG2* promoters. No differences were observed for the other globin genes, including *HBB*, or for the distal enhancers of the locus control region (LCR) (Fig. 3d, Suppl Table 2d). Of note, while the HPFH samples showed increased accessibility of the *HBG1/2* promoters compared to control samples, a further subdivision of the HPFH samples into HbF levels above or below 28% yielded differential accessibility of 99 peaks between the two groups, but these did not include the *HBG1/2* promoters (Fig. 3d, Suppl Table 2e). This raised the question whether chromatin accessibility of differentially expressed genes other than *HBG1/2* is affected by *KLF1* haploinsufficiency.

### Chromatin accessibility at KLF1 target genes

**Figure 4:**
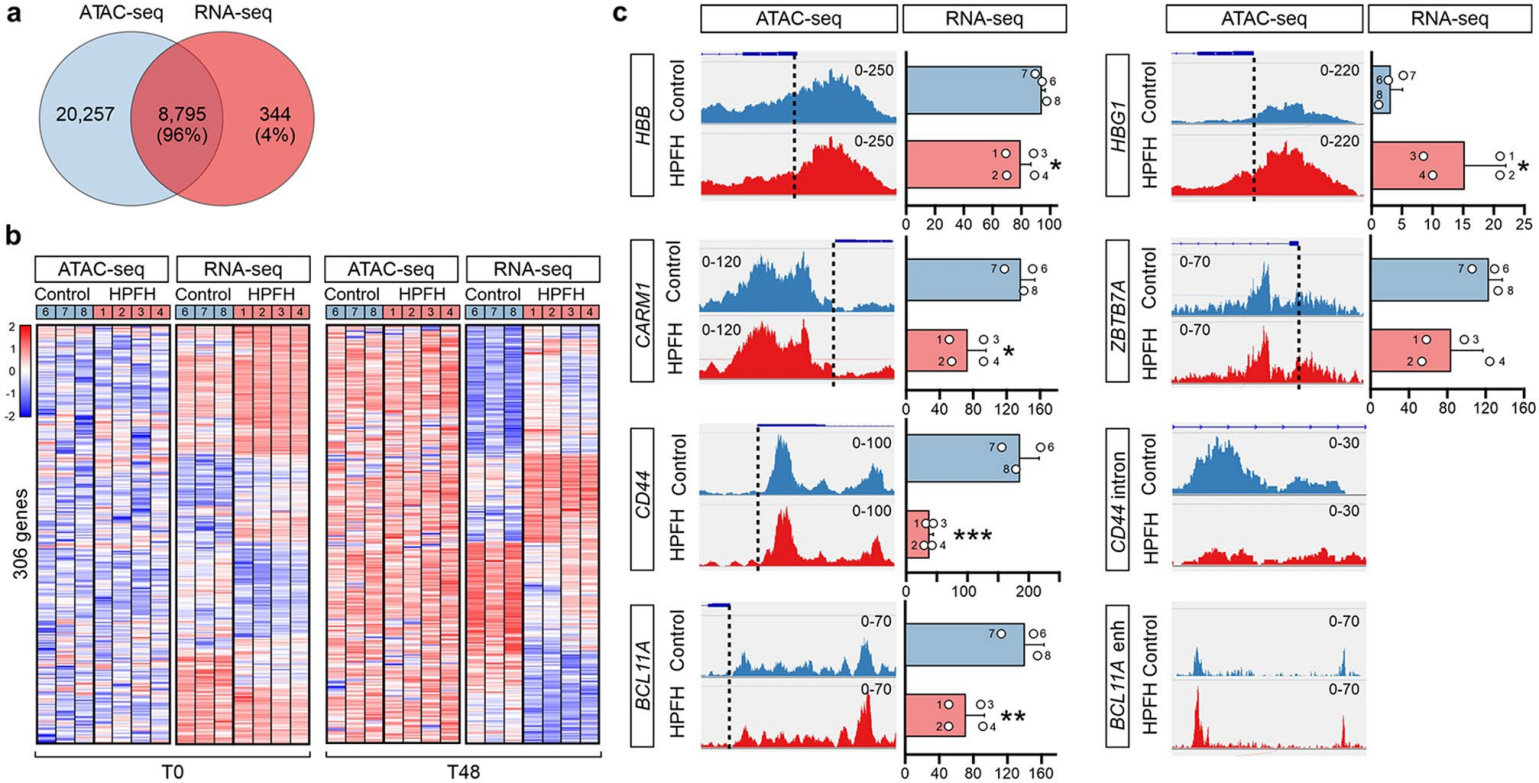
Integration of ATAC-seq and RNA-seq analysis. (**a**) Venn-diagram displaying overlap between ATAC-seq and mRNA-seq at T48. Counts represent the number of individual genes above minimum threshold for mRNA expression (> 3 CPM) or open chromatin (> 1 CPM). (**b**) Heatmaps showing normalized mRNA expression and chromatin accessibility at T0 and T48 for differentially expressed genes between control and HPFH samples. (**c**) Chromatin accessibility and mRNA expression of differentially expressed genes at T48. For each gene the genome track shows chromatin accessibility. Dotted lines indicate transcription start sites. Bar plots indicate mRNA expression values. The individuals from whom the samples were derived are indicated (Table 1). CPM: counts per million mapped reads; enh = erythroid enhancer in intron 2 of the *BCL11A* gene. mean + /- SD; *P < 0.05, **P < 0.01, ***P < 0.01, Students T-test.

Whereas 96% of the 9136 expressed genes and 90% of differentially expressed genes identified by RNA-seq also contained one or more ATAC peaks (Fig. 4a), only 27 of the 344 differentially expressed genes displayed altered chromatin accessibility (Suppl Fig. 4d, Suppl Table 2a). The majority of differentially expressed genes had more ATAC signal at T48 compared to T0 indicating increased chromatin accessibility upon differentiation, regardless of sample genotype or direction of differential gene expression (Fig. 4b). As discussed above, the *HBG1/2* promoters displayed increased accessibility in HPFH samples, which was paired with increased γ-globin RNA expression. In contrast, the *HBB* promoter, known to bind KLF1^37, 38^, did not show reduced accessibility paired with reduced β-globin RNA expression (Fig. 4c). Similar to *HBB*, KLF1 target genes that were previously identified by ChIP such as *BCL11A* and *CARM1* showed differential RNA expression, but no differential chromatin accessibility. In contrast, *CD44* showed both reduced accessibility and reduced expression (Fig. 4c). However, here differential accessibility was found in the first intron, while KLF1 binding was reported at the TSS^5^. Together this suggests that *KLF1* haploinsufficiency is not resulting in an altered chromatin accessibility of its target genes. Rather, the increased fraction of genes with BCL11A and LRF binding sites among the differentially expressed genes suggests that indirect regulation by KLF1 is an important mechanism in those cases where both gene expression and chromatin accessibility were altered.

### Specific SNPs within the promoter of *KLF1* may influence promoter activity

**Figure 5:**
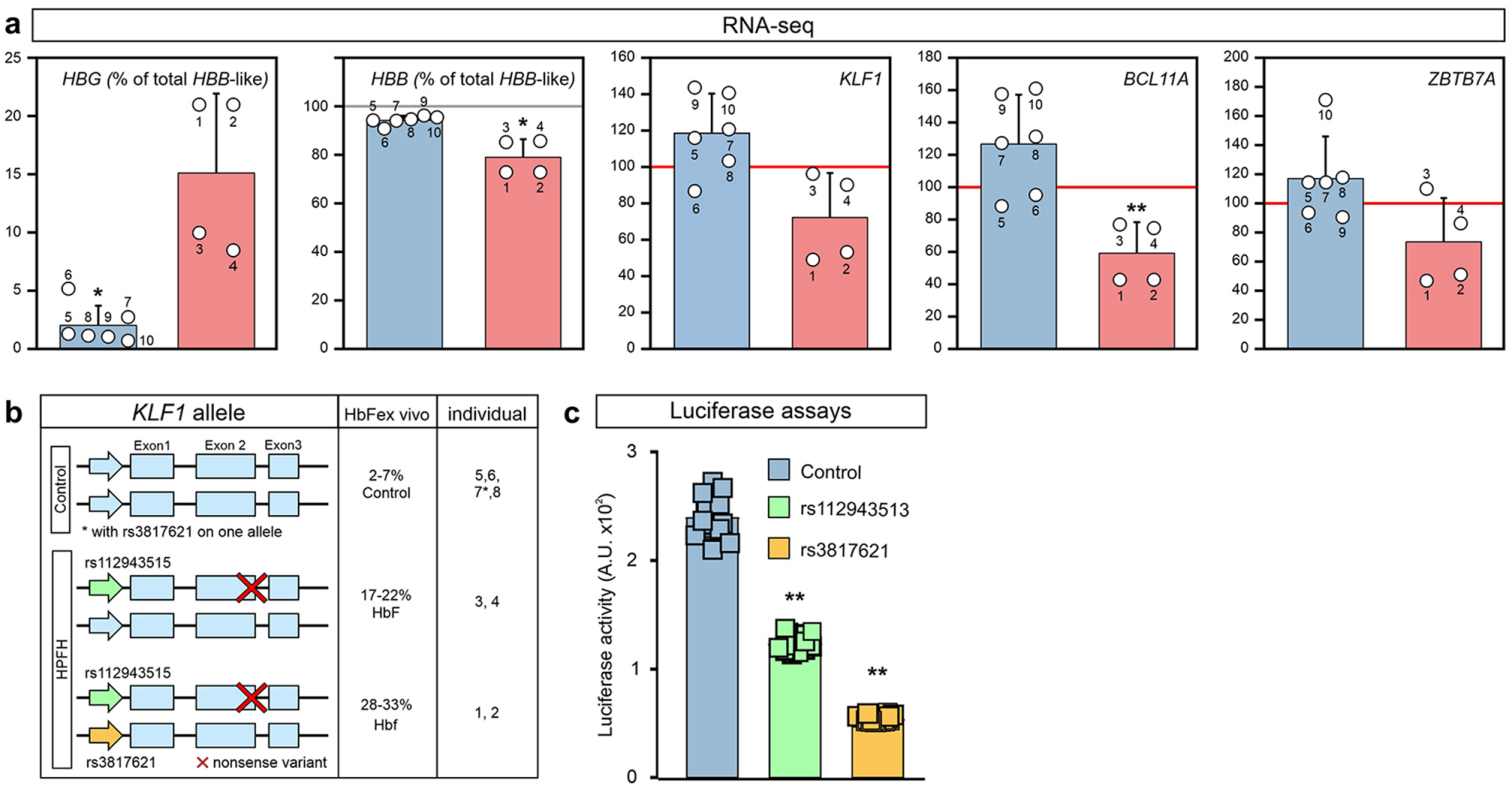
Variant *KLF1* alleles may affect promoter activity. (**a**) Expression of selected genes assessed by RNA-seq at T48. For *HBG1/2* (*HBG*) and *HBB*, expression is calculated as percentage of expression of all *HBB*-like genes (*HBE1* + *HBG1* + *HBG2* + *HBD* + *HBB*). For *KLF1*, *BCL11A* and *ZBTB7A*, the average expression level of all samples was normalized to 100 (red line). The individuals from whom the samples were derived are indicated (Table 1). (**b**) Schematic representation of genomic layout of the *KLF1* alleles present in the individuals studied here. A promoter variant, rs381762, is present in one of the control individuals (individual 7) and two HPFH individuals (individuals 1, 2). The red cross indicates the *KLF1* p.K288X mutation. (**c**) Dual Luciferase assays in K562 cells with reporter plasmids driven by *KLF1* promoter variants. A.U.: arbitrary units of luciferase activity, corrected for transfection efficiency. *P < 0.05, **P < 0.01, Students T-test. Error bars indicate SD.

Next, we asked if variation in residual *KLF1* expression might be linked to variability in HbF levels between HPFH cultures. In the RNA-seq data we observed an inverse correlation between *HBG1/2* and *KLF1* expression (Fig. 2c and Fig. 5a). In contrast, previously identified KLF1 targets *HBB*, *BCL11A*, *ZBTB7A*, *PLEK2*, and *CARM1* displayed a positive correlation with KLF1 expression, while *SOX6* expression was constant and *MYB* expression very variable (Figs. 2c, 5a, Suppl Fig. 5). Within the *KLF1* promoter several SNPs that may influence *KLF1* expression have been annotated^39^. Sanger sequencing showed that control individual 7 and HPFH individuals 1 and 2 carried the minor allele of a SNP, rs3817621, located at position -251 in the *KLF1* promoter (Fig. 5b, Suppl Table 1). The minor allele of SNP rs112943515 further discriminated between HPFH and control samples, as it was linked to the *KLF1* p.K288X allele (Fig. 5b, Suppl Table 1). To test whether rs3817621 and rs112943515 might affect promoter activity, luciferase reporter experiments using the *KLF1* promoter were performed in K562 cells. Promoters containing the minor alleles of rs3817621 or rs112943515 showed reduced luciferase expression compared to the wildtype *KLF1* promoter, with rs3817621 having the strongest effect (Fig. 5c). For both rs1129435 and rs3817621 this difference reached statistical significance (*P* < 0.01; Students T-test) when compared to the control experiments. The HPFH samples with high ex vivo HbF levels (Table 1, Suppl Table 1) carry the *KLF1* minor allele of promoter variant rs3817621 which may render the *KLF1* promoter less active (Fig. 5c). However, individual 2 has the lowest in vivo HbF level (1.3%) of the four HPFH subjects studied here, while individual 1 has the highest (12.3%, Table 1, Suppl Table 1).Thus, presence of the rs3817621 minor allele alone is not sufficient to explain the variable in vivo HbF levels in Maltese HPFH individuals. Collectively, based on our ex vivo data we hypothesize that, on top of the effect of haploinsufficiency, variation in wildtype *KLF1* expression affects HbF levels via modulation of *BCL11A* and *ZBTB7A* expression.

## Discussion

Reduced expression of KLF1 decreases expression of the *HBG1/2* repressors BCL11A and LRF, thus enhancing HbF expression^2, 4, 5^. There are, however, considerable differences in HbF levels between carriers of the *KLF1* p.K288X haploinsufficiency allele, prompting us to find an explanation for this variability. We found that a SNP (rs3817621) in the promoter of the remaining wildtype *KLF1* allele, which reduces *KLF1* promoter activity in luciferase assays, correlated with high HbF levels in cultured erythroid progenitors derived from Maltese *KLF1* p.K288X carriers. The rs3817621variant is present in HPFH individuals 1 and 2, displaying the highest ex vivo HbF levels. However, the in vivo HbF level of HPFH individual 2 is the lowest (1.3%) of the four HPFH individuals included here (Table 1, Suppl Table 1). Thus, our studies do not allow extrapolation to the role of rs3817621 in steady-state erythropoiesis in vivo. We note that erythroid progenitor cultures are thought to represent stress erythropoiesis^32^, which might explain the discrepancy between the in vivo and ex vivo observations. SNP rs3817621 (minor allele frequency 0.234) is not associated with the typical reduction in Lutheran and Indian blood groups or altered globin expression caused by *KLF1* variants^9^. Intriguingly, rs3817621 was found to be associated with red blood cell indices in a population study^39, 40^, supporting the notion that minor alleles may affect the activity of the *KLF1* promoter in vivo. From our experimental data we surmise that subtle variations in expression of the remaining wildtype *KLF1* allele affect HbF levels through differential expression of the two major *HBG1/2* repressors BCL11A and LRF. This is consistent with previous work establishing that expression of these two repressors is under direct control of KLF1^3–5, 10^. We propose that the HbF level is the resultant of the compound effect of the activity of the remaining wildtype *KLF1* allele and the p.K288X *null* variant on the other *KLF1* allele. These individuals should therefore be considered as compound heterozygote carriers of *KLF1* variants, as their phenotype extends beyond that expected for *KLF1* haploinsufficiency. Finally, in agreement with our previous microarray RNA expression data^4^ western blot analysis showed reduced expression of KLF1 in proerythroblasts derived from individuals carrying the p.K288X *KLF1* allele. However, a further differential KLF1 protein expression between the individual HPFH cases could not be demonstrated by western blotting (data not shown). We believe that this reflects a technical limitation of quantitative western blot analysis due to the error margin of this technique, which may therefore fail to reliably detect micro-variation in KLF1 expression.

### Increased chromatin accessibility of the *HBG1/2* promoters

In erythroid progenitors from *KLF1* p.K288X-bearing individuals, the HPFH phenotype is associated with increased chromatin accessibility at the *HBG1/2* promoters. Notably, this increased promoter accessibility is an exception, as this is not observed for promoters of the vast majority of the 344 deregulated genes observed between control and HPFH erythroid cells. This overwhelming absence of locally altered chromatin accessibility in HPFH erythroblasts shows there is no major direct impact of reduced KLF1 levels on chromatin accessibility of its target genes. This is supported by cross-referencing the genes with differential expression and differential chromatin accessibility against datasets from published ChIP-seq experiments^2, 5^. These genes showed enrichment for binding sites of BCL11A and LRF. In the context of HbF regulation, this is consistent with the hypothesis that reduced KLF1 expression results in downregulation of many of its target genes, including genes encoding the *HBG1/2* repressors LRF and BCL11A. These repressors directly bind and repress the *HBG1/2* promoters in adult erythroid cells^1, 3^. Presumably, diminished expression of LRF and BCL11A results in reduced binding to the *HBG1/2* promoters. Complementary reduced interaction of the *HBB* promoter with the LCR, previously described in the context of *Klf1 null* mice^37^, favors activation of the accessible *HBG1/*2 promoters. We propose that this dual role of KLF1, along with micro-variation in KLF1 expression between HPFH individuals, explains a significant part of the variable penetrance of HbF expression levels associated with *KLF1* haploinsufficiency.

### *KLF1* haploinsufficiency does not affect erythroid chromatin accessibility broadly

In addition to the *HBG1/2* promoters there is a limited set of ATAC peaks, consisting of less than 2% of the total, that also show differential accessibility in the HPFH cells. Although most of the genes associated with these regions do not display differential expression, it remains possible that these regions act as distal regulatory elements for more remotely located genes. Since erythroid differentiation is accompanied by broad scale chromatin reorganization^35, 41^, the limited set of regions changed during *KLF1* haploinsufficiency may potentially interfere with differentiation. However, cell morphology and expression of differentiation markers are largely unperturbed in *KLF1* haploinsufficiency indicating that the remaining KLF1 expression is sufficient to keep erythroid chromatin accessibility compatible with terminal erythroid differentiation. Reduced expression of the nuclear exportin XPO7, which in turn affects global chromatin condensation^42, 43^, provides an alternative explanation for the increased chromatin accessibility. Similar to the *HBB* locus*,* regulation of *Xpo7* expression by KLF1 ensues via a chromatin looping mechanism in mice^42^. Possibly, this mode of regulation renders genes particularly susceptible to the effects of reduced KLF1 levels. This point deserves future investigation of long-range interactions using chromosome conformation capture techniques^44, 45^.

### Phenotypic variability associated with haploinsufficiency for transcription regulators

The notion of variable penetrance resulting from mutated alleles was addressed in a study on the roundworm *Caenorhabditis elegans*. Expression of individual transcripts was counted in a small three-layer regulatory network^46^. Mutations to the initiating node of the network caused highly variable expression of the intermediate nodes and resulted in bimodal expression of the most downstream genes. For different mutant alleles the threshold required for the downstream effects shifted, which was proposed as an explanation for incomplete penetrance. In the context of Maltese HPFH, we propose that micro-variation in expression of the wildtype *KLF1* allele alters direct and indirect regulation of globin gene expression from the *HBB* locus. Thus, the variable penetrance of high HbF expression in *KLF1* haploinsufficiency fits very well with the idea that micro-variation in expression of a critical factor can be amplified in a regulatory network. In summary, we propose that micro-variations in KLF1 levels are a major source of variable HbF expression observed in *KLF1* haploinsufficiency. Our findings have broader implications for understanding the phenotypic variability associated with mutations in other hematopoietic transcription factors, such as RUNX1, EP300 and GATA2. For example, variations in mono-allelic *GATA2* expression were recently shown to reduce penetrance in patients with hereditary *GATA2*-mutated MDS/AML^47^. Similarly, micro-variations in *RUNX1* and *EP300* expression might help explain the range of phenotypes observed in response to mutations in these factors^48–50^. Such cases of differential penetrance illustrate the importance of extended genotypic screening of transcription factor loci, in order to improve prognostic and therapeutic strategies in the event of haploinsufficiency.

## Methods

### Erythroid cultures

Blood samples were harvested following informed consent in accordance with the Declaration of Helsinki, under University of Malta approval (FREC 45/2014), and from anonymous Dutch blood donors. Written informed consent to extract and use human blood was approved by EAR (Ethical Advisory Council Sanquin, The Netherlands) and according to the guidelines of NetCord FACT. Viable cells of the Maltese donors have been deposited in the BioBank of the University of Malta. Erythroid cultures were started as described previously^31, 32^. For details see Supplementary Materials and Methods.

### Flow cytometry

Cells were fixed with 0.025% glutaraldehyde, 0.5% paraformaldehyde and permeabilized with 0.05% NP40 (Sigma Aldrich), followed by incubation with primary antibodies: CD71-vio-blue421, CD235a-PE, CD44-APC, HbA-PE, HbF-APC. For details see Supplementary Materials and Methods.

### Hemoglobin content and cell morphology

Aliquots (1 × 10^5^ cells) were analyzed for hemoglobin content by spectrophotometry as described^51^. Cell morphology was analyzed in cytospins stained with Giemsa and neutral benzidine^52^, using an Olympus BX40 microscope (40 × objective, NA 0.65), and a Leica DM-2500digital camera.

### HPLC

1 × 10^7^ cells were collected and analyzed for hemoglobin expression by high-performance cation-exchange liquid chromatography (HPLC) on Waters Alliance 2690 equipment. The column was purchased from PolyLC^53^.

### Real-time quantitative PCR

Samples were collected from expanding (T0) and differentiating (T48) erythroid cultures. RNA was isolated using TRIzol RNA isolation reagents (ThermoFisher Scientific) and used for RT-qPCR analysis as described in Supplementary Materials and Methods.

### Next generation sequencing

For RNA sequencing libraries were prepared using the TruSeq Stranded mRNA library kit (Illumina) according to manufacturer’s instructions. For ATAC sequencing samples were prepared according to^29^ using the Nextera DNA transposase kit. Sequencing was performed on an Illumina HiSeq2500 sequencer. The data has been deposited in the European Nucleotide Archive (https://www.ebi.ac.uk/ena) under accession number PRJEB31712. Further details and data analysis are described in Supplementary Materials and Methods.

### Promoter assays

The plasmids containing *KLF1* promoter variants (rs112943513 or rs3817621) in the pGL4.10 *Photinus pyralis* luciferase reporter constructs were generated by Mutagenex, Inc. The pRL-TK *Renilla reniformis* luciferase vector was used as an internal control for transfection efficiency and promoter activity. The reporters were transfected into K562 cells and dual luciferase activity was measured according to the manufacturer’s guidelines (Promega). For dual luciferase assays, statistical significance was calculated by performing One Sample Students T-tests for at least three independent experiments. Further details of the promoter constructs are described in Supplementary Materials and Methods.

## Supplementary Information

Supplementary Information 1.

Supplementary Information 2.
