# Supplementary material for: Epigenomic analysis of KLF1 haploinsufficiency in primary human erythroblasts
Source: Sci Rep. 2022 Jan 10;12:336. doi: 10.1038/s41598-021-04126-6 (PMC8748495; doi:10.1038/s41598-021-04126-6)
Supplement: Supplementary file 1 — Supplementary Information 1. [file 41598_2021_4126_MOESM1_ESM.pdf]

Epigenomic analysis of *KLF1* haploinsufficiency in primary human erythroblasts

Steven Heshusius, Laura Grech, Nynke Gillemans, Rutger W. W. Brouwer, Xander T. den Dekker, Wilfred F.J. van IJcken, Ben Nota, Alex E. Felice, Thamar B. van Dijk, Marieke von Lindern, Joseph Borg, Emile van den Akker and Sjaak Philipsen

Supplementary Materials and Methods
Supplementary Table 1
Legend to Supplementary Table 2
Supplementary Figures

Supplementary Materials and Methods

Erythroid cultures

Erythroblasts were expanded from peripheral blood mononuclear cells in Cellquin culture medium (Sanquin) supplemented with stem cell factor (SCF, 293T producer cell line supernatant equivalent to 100ng/ml), EPO (2 U/ml, ProSpec) and dexamethasone (Dex, 1µM, Sigma Aldrich) for ten days [1,2]. Medium was refreshed every other day, cells were counted and maintained at 0.7-2*10^6 cells/ml (CASY Model TCC, Innovatis AG). Erythroblast differentiation was induced in medium with high EPO (10 U/ml) and 5% human plasma (Sanquin) [1]. At start of differentiation cells were seeded at 2*10^6 cells/ml, and the medium was partly replenished every other day for the first four days of differentiation.

Flow cytometry

Cells were fixed with 0.025% glutaraldehyde, 0.5% paraformaldehyde and permeabilized with 0.05% NP40 (all from Sigma Aldrich), followed by incubation with primary antibodies. Details of the antibodies are listed below. Samples were measured on a Becton-Dickinson LSR II flowcytometer with HTS plate reader (Becton-Dickinson Bioscience) and analyzed using FlowJo software (Becton-Dickinson, v10.6)

Antibody	Dilution	Fluorochrome	Producer	Catalog #
CD71 /Transferrin receptor	1:200	Vio-blue 421	Miltenyi	130-101-631
CD235 /Glycophorin A	1:2500	PE	Acris	DM066P
CD44	1:200	FITC	eBioscience	11-0441-81
HbA /Adult hemoglobin	1:1000	PE	Santa Cruz	Sc-21757
HbF /Fetal hemoglobin	1:1000	APC	Invitrogen	HBF-1
IgG1 k control	1:200	PB	Biolegend	400172
IgG1 k control	1:200	FITC	Biolegend	M1453
IgG1 control	1:1000	PE	Diaclone	857.072.010
IgG1 k control	1:1000	APC	eBioscience	P3.6.2.8.1

Real-time quantitative PCR

cDNA was synthesized with the QuantiTect reverse transcription kit (QIAGEN, #205313) according to the manufacturers' instructions. Quantitative PCR was performed on a StepOnePlus Real-Time PCR system (ThermoFisher Scientific, #4376600) with Power SYBR green master mix (ThermoFisher Scientific, #4367659) and 1µM primers in a final volume of 20µl. The following program was used: 10min at 95 °C, followed by 40 cycles x 15sec at 95 °C and 1min at 60 °C. Ct-values were normalized against housekeeping genes 18S and HPRT. Graphpad Prism V7.04 (GraphPad Software) was used for statistic testing and visualization of fold-change mRNA expression. Primers used for RT-qPCR are listed below.

Primer	Sequence (5’ – 3’)
HBB fw	aca gcc acc act ttc tga t
HBB rv	agc tgc act gtg aca agc tg
HBG1/2 fw	aaa cgg tca cca gca cat tt
HBG1/2 rv	gaa ggt gct gac ttc ctt gg
KLF1 fw	tcc cac ctg aag gcg cat ct
KLF1 rv	cag ctg gtc aga gcg cga aa
BCL11A fw	gcc cca aac agg aac aca ta
BCL11A rv	ttg caa gag aaa cca tgc ac
ZBTB7A fw	gct gcg gca cct tta aga ca
ZBTB7A rv	gcg tca ctg ccc cta ca
18S fw	cac ggc cgg tac agt gaa ac
18S rv	aga gga gcg agc gac caa
GAPDH fw	cat cac gcc aca gtt tcc
GAPDH rv	tcc cat cac cat ctt cca

RNA library preparation for sequencing (RNA-seq)

RNA-Seq library was prepared for analysis according to the Illumina TruSeq stranded mRNA protocol (www.illumina.com). Briefly, 200 ng of total RNA was purified using poly-T oligo-attached magnetic beads to end up with poly-A containing mRNA. The poly-A tailed mRNA was fragmented and cDNA was synthesized using SuperScript II and random primers in the presence of Actinomycin D. cDNA fragments were end repaired, purified with AMPure XP beads, A-tailed using Klenow exo-enzyme in the presence of dATP. Paired-end adapters with dual index (Illumina) were ligated to the A-tailed cDNA fragments and purified using AMPure XP beads. The resulting adapter-modified cDNA fragments were enriched by PCR using Phusion polymerase as followed: 30 s at 98°C, 15 cycles of (10 s at 98°C, 30 s at 60°C, 30 s at 72°C), 5 min at 72°C. PCR products were purified using AMPure XP beads and eluted in 30 µl of resuspension buffer. One microliter was loaded on an Agilent Technologies 2100 Bioanalyzer using a DNA 1000 assay to determine the DNA concentration and for library quality check.

Sample preparation for ATAC-seq

The Assay for Transposase Accessible Chromatin coupled to high-throughput sequencing (ATAC-seq) was performed as previously described [3] with slight modifications. Briefly, approximately 50,000 single cells were resuspended in 50 µl of cold lysis buffer (10 mM Tris-HCl (pH 7.4) 10 mM NaCl, 3 mM MgCl2, 0.1% IGEPAL CA-630). Immediately after lysis, nuclei were centrifuged at 500xg for 10 min at 4°C. Nuclei were re-suspended in 25 µl 2x TD buffer (Illumina), 2.5 µl TDE1 (transposase, Illumina) and 20 µl nuclease-free water, and incubated for 30 min at 37°C. Afterward, the sample was purified using the MinElute PCR Purification Kit (QIAGEN) according to the manufacturer’s protocol and eluted with 10 µl elution buffer. Samples were amplified using the NEBNext High Fidelity PCR master mix (New England Biolabs) and afterwards purified using a MinElute PCR purification kit (Qiagen) according to

the manufacturer's instructions. One microliter was loaded on an Agilent Technologies 2100 Bioanalyzer using a DNA 1000 assay to determine the DNA concentration and for library quality check.

Bridge amplification and sequencing by synthesis for RNA-seq and ATAC-seq

Cluster generation was performed according to the Illumina TruSeq SR Rapid Cluster kit v2 (cBot) Reagents Preparation Guide (www.illumina.com). Briefly, for sequencing libraries were pooled together to get a stock of 10 nM. One microliter of the 10 nM stock was denatured with NaOH, diluted to 10 pM and hybridized onto the flow cell. The hybridized products were sequentially amplified, linearized and end-blocked according to the Illumina Single Read Multiplex Sequencing user guide. After hybridization of the sequencing primer, sequencing-by-synthesis was performed using the HiSeq 2500 with either a single read 50-cycle (for RNA-seq) or paired end 50-cycle protocol followed by dual index sequencing (for ATAC-seq). Both RNA and ATAC libraries were sequenced to a depth of 15×10^6 clusters. The raw data (fastq files) has been deposited in the European Nucleotide Archive (https://www.ebi.ac.uk/ena) under accession number PRJEB31712.

Data analysis

RNA-seq reads were mapped to GRCh38.v85 using STAR. Lowly expressed mRNA (<3 counts per million mapped reads in less than 4 samples) were filtered prior to differential expression analysis with EdgeR package. Quasi-maximum-likelihood F-test was used for testing, with false discovery rate (FDR) multiple testing correction. FDR < 0.05 was chosen as threshold for differential expression [4]. For ATAC-seq reads were mapped to GRCh38.v85 using Bowtie2 [5]. Low abundant regions (<1 counts per million mapped reads in less than 3 samples) were filtered prior to differential analysis in EdgeR. Log-odds ratio test with FDR < 0.05 as threshold for differential expression. For both RNA-seq and ATAC-seq three comparisons were tested: T0 vs T48 with paired samples, T0 HPFH vs. control and T48 HPFH vs control. ATAC-seq peaks were called with MACS2 peak calling algorithm discriminating ATAC peaks over background signal (q-value < 0.05) [6]. The ATACseqQC R-package was used to plot insert size periodicity and to classify reads belonging to mono- or multiple-nucleosome-free regions prior to plotting position relative to transcription start sites [7].

Promoter assays

The *KLF1* promoter region used encompassed chr19: 12887181 to 12888463 (-), version GRCh38.p13 of the human genome. EcoRV was used for cloning in the pGL4.10 *Photinus pyralis* luciferase reporter vector.

rs112943513: NC_000019.10:g.12888273G>T, MAF (T) 0.011 (ALFA)

CACCGCACCTGGCCTTGTTTTTGTTTGTGTCACCCAGGTTGATCTGCCAGGAATAAAGAT
GGCCACCCAACTTTTTTTTTTTTCCAGCGACAGAGTCTTGCTTTCTGTGCCCCAGGCTGG
ACTGCAGTGGTGTGATTATAGTTCAGTGCAGTCCCGACCTCCTGGGCTCAGGCAATCCTC
CCACCTCAGCC**C/A**TCCTGAGTAGCTGGGACTATGGGCATGCACCACCACTCACAGCTTTTT
GTTGGAGAGATAGGTTTTATGTTGCCCAGGCTGGTCTTGAAATCCTGGTGTCAAGTGATC
CTCCTGCCTCAGTCTTCCAATGAGTGACTATGAGTTATGTGTCTGTAATACGTATCCATG
TCCCCTTCCCAGGCTCCCAGGGCACCAGAGTGGAGGTTCCTGTTGTAGAAACTCAGATCC
TCTCCTCATGTTGGGCAGAATCAAGGAGCAGCCAGGCCCAGAGCCAGGGCACTGGTCTCC
TGCAGGTCAGGTACTGCCTGTGGCTTGATCCAACGGTCCTATCCCACCCAGGAGGAGAGA
GGGTCACTTTTCCCTTGGCTGCCCCCATCGCACTAAAGCAGCTGGCACTGAACCAGCCTC
CATGCAGTCCCATGCAGTGCCACCCAAGGGTCCCCAGTAGACAATGGTGGGCCAGTTGTC
AGGGGCTTCCTCCTGCTGCAGGGCTGAGACCCTGGGAGGTCCCAACCCAGGCAAATTGAA
CGCCAGGCTAATTTGAAGACCCAACTCCCAGCCCTCCCCTTCACCGGAGGACAGAGCTCT
AGCTGGCCTGGGCCCCCACCTGATAGCAGCCTCCAACGTCTGGGGTGTCTGATAATGCTT
GGCGGGGAGCTCGTGCCAAGTCCCGCCATCAGCACGGTTGTTGCTGTTTACTGGGGAGGG
GGAGGGCTGTGGAGCCTCAATCAGGGGGACAGGGGGTCCCACAGCTTCTTCCCAGAATAC
CCTTTCTGCCTTTTCCAGGAAAGTTAACTGAGGGAAGACCCCCAAGTCTCTCCTTCTTTG
GAGACCCAATGTCTGTTTTTACCCAGCACCTGGACCCTCAAACCCTGAACCCCCCAACCC
TTGATATTTGACTTGGCTTTGGACACAGGGTTAGTCTTTAACCCCAGCCCCAGACAGGCC
AACGTGAAGTTTGTGCCCCAGAAACAGTGCCCCCCCGCCGCCTTGCCTTGCTTTGCCTTA
TCAGAGGCTGCAGCCAATCAGCTAAGGACAGAGAGGAGCCCTCGAAGGGGCTATCACAGC
CTCAGAGTTCACGAGGCAGCCGA**GATATC**AAGATCTGGCCTCGGCGGCCAAGCTTGGCAA
TCCGTACTGTTGGTAAAGCCACC**ATG**GAAGAT... Luciferase coding region

rs3817621: NC_000019.10:g.12887391G>C, MAF (C) 0.234 (ALFA)

CACCGCACCTGGCCTTGTTTTTGTTTGTGTCACCCAGGTTGATCTGCCAGGAATAAAGAT
GGCCACCCAACTTTTTTTTTTTTCCAGCGACAGAGTCTTGCTTTCTGTGCCCCAGGCTGG
ACTGCAGTGGTGTGATTATAGTTCAGTGCAGTCCCGACCTCCTGGGCTCAGGCAATCCTC
CCACCTCAGCCCTCCTGAGTAGCTGGGACTATGGGCATGCACCACCACTCACAGCTTTTT
GTTGGAGAGATAGGTTTTATGTTGCCCAGGCTGGTCTTGAAATCCTGGTGTCAAGTGATC
CTCCTGCCTCAGTCTTCCAATGAGTGACTATGAGTTATGTGTCTGTAATACGTATCCATG
TCCCCTTCCCAGGCTCCCAGGGCACCAGAGTGGAGGTTCCTGTTGTAGAAACTCAGATCC
TCTCCTCATGTTGGGCAGAATCAAGGAGCAGCCAGGCCCAGAGCCAGGGCACTGGTCTCC
TGCAGGTCAGGTACTGCCTGTGGCTTGATCCAACGGTCCTATCCCACCCAGGAGGAGAGA
GGGTCACTTTTCCCTTGGCTGCCCCCATCGCACTAAAGCAGCTGGCACTGAACCAGCCTC
CATGCAGTCCCATGCAGTGCCACCCAAGGGTCCCCAGTAGACAATGGTGGGCCAGTTGTC
AGGGGCTTCCTCCTGCTGCAGGGCTGAGACCCTGGGAGGTCCCAACCCAGGCAAATTGAA
CGCCAGGCTAATTTGAAGACCCAACTCCCAGCCCTCCCCTTCACCGGAGGACAGAGCTCT
AGCTGGCCTGGGCCCCCACCTGATAGCAGCCTCCAACGTCTGGGGTGTCTGATAATGCTT
GGCGGGGAGCTCGTGCCAAGTCCCGCCATCAGCACGGTTGTTGCTGTTTACTGGGGAGGG
GGAGGGCTGTGGAGCCTCAATCAGGGGGACAGGGGGTCCCACAGCTTCTTCCCAGAATAC
CCTTTCTGCCTTTTCCAGGAAAGTTAACTGAGGGAAGACCCCCAAGTCTCTCCTTCTTTG
GAGACCCAATGTCTGTTTTTACCCAGCACCTGGACCCTCAAACCCTGAACCCC**C/G**CAACCC
TTGATATTTGACTTGGCTTTGGACACAGGGTTAGTCTTTAACCCCAGCCCCAGACAGGCC
AACGTGAAGTTTGTGCCCCAGAAACAGTGCCCCCCCGCCGCCTTGCCTTGCTTTGCCTTA
TCAGAGGCTGCAGCCAATCAGCTAAGGACAGAGAGGAGCCCTCGAAGGGGCTATCACAGC
CTCAGAGTTCACGAGGCAGCCGA**GATATC**AAGATCTGGCCTCGGCGGCCAAGCTTGGCAA
TCCGTACTGTTGGTAAAGCCACC**ATG**GAAGAT... Luciferase coding region

References

1 Heshusius, S. *et al.* Large-scale in vitro production of red blood cells from human peripheral blood mononuclear cells. *Blood Adv* **3**, 3337-3350, doi:10.1182/bloodadvances.2019000689 (2019).

2 van den Akker, E., Satchwell, T. J., Pellegrin, S., Daniels, G. & Toye, A. M. The majority of the in vitro erythroid expansion potential resides in CD34(-) cells, outweighing the contribution of CD34(+) cells and significantly increasing the erythroblast yield from peripheral blood samples. *Haematologica* **95**, 1594-1598, doi:10.3324/haematol.2009.019828 (2010).

3 Buenrostro, J. D., Giresi, P. G., Zaba, L. C., Chang, H. Y. & Greenleaf, W. J. Transposition of native chromatin for fast and sensitive epigenomic profiling of open chromatin, DNA-binding proteins and nucleosome position. *Nat Methods* **10**, 1213-1218, doi:10.1038/nmeth.2688 (2013).

4 Robinson, M. D., McCarthy, D. J. & Smyth, G. K. edgeR: a Bioconductor package for differential expression analysis of digital gene expression data. *Bioinformatics* **26**, 139-140, doi:10.1093/bioinformatics/btp616 (2010).

5 Langmead, B. & Salzberg, S. L. Fast gapped-read alignment with Bowtie 2. *Nat Methods* **9**, 357-359, doi:10.1038/nmeth.1923 (2012).

6 Zhang, Y. *et al.* Model-based analysis of ChIP-Seq (MACS). *Genome Biol* **9**, R137, doi:10.1186/gb-2008-9-9-r137 (2008).

7 Ou, J. *et al.* ATACseqQC: a Bioconductor package for post-alignment quality assessment of ATAC-seq data. *BMC Genomics* **19**, 169, doi:10.1186/s12864-018-4559-3 (2018).

8 Borg, J. *et al.* Haploinsufficiency for the erythroid transcription factor KLF1 causes hereditary persistence of fetal hemoglobin. *Nat Genet* **42**, 801-805, doi:10.1038/ng.630 (2010).

Supplementary Table 1
Additional data on Maltese HPFH pedigrees FamD and FamF

Family	Member	Number	RNA-seq	ATAC-seq	Gender	Hb g/dl	MCV	F cells %	HbA2 %	HbF mg/dl	HbF %	HbF % *	rs112943513 promoter variant	rs3817621 promoter variant	rs267607202 p.K288X
FamD	I-1				M	15.1	85	16	3.4	260	1.7		GT	GG	TA
FamD	II-2	2	yes	yes	M	14.6	83	15	3.3	230	1.6	1.34	GT	GC	TA
FamD	II-1	7	yes	yes	M	14.5	88	2	3.0	80	0.6	0.17	GG	GC	TT
FamD	I-2				F	11.4	80	2	2.7	40	0.4		GG	GC	TT
FamF	II-5	1	yes	yes	F	12.4	76	54	2.6	2480	20.0	12.32	GT	GC	TA
FamF	II-1				M	14.5	75	50	2.6	2465	17.0		GT	GC	TA
FamF	III-10				F	13.8	77	40	2.9	1242	9.0		GT	GG	TA
FamF	III-16	3	yes	yes	F	12.9	77	39	3.1	1135	8.8	7.26	GT	GG	TA
FamF	III-6				M	12.9	76	36	3.4	1071	8.3		GT	GG	TA
FamF	III-2				F	13.8	74	26	3.3	828	6.0		GT	GG	TA
FamF	III-15	4	yes	yes	F	12.8	74	29	3.5	717	5.6	3.80	GT	GG	TA
FamF	II-7				F	13.8	73	17	3.5	483	3.5		GT	GC	TA
FamF	III-9				F	11.9	76	26	3.4	405	3.4		GT	GG	TA
FamF	III-4				M	13.7	77	16	3.5	411	3.0		GT	GG	TA
FamF	IV-5				M	12.1	79	19	2.5	296	2.4		GG	GC	TT
FamF	III-1				M	14.8	82	12	3.3	281	1.9		GG	GC	TT
FamF	III-7				F	12.8	83	14	2.7	230	1.8		GG	GC	TT
FamF	III-3				F	12.4	84	20	3.2	223	1.8		GG	GC	TT
FamF	III-11				F	12.2	83	10	2.8	122	1.0		GG	GC	TT
FamF	II-6	5	yes	-	M	14.8	85	2	3.2	59	0.4	0.16	GG	GG	TT
FamF	III-14	6	yes	yes	M	14.8	86	3	2.7	59	0.4	0.60	GG	GG	TT
FamF	III-17				M	12.4	86	0	2.8	40	0.3		GG	GG	TT
FamF	II-2				F	12.6	85	1	3.5	38	0.3		GG	GG	TT

* At time of blood collection for cell culture.

See Supplementary Figure 1 for trees of the Maltese HPFH pedigrees FamD and FamF.

Supplementary Table 2 (excel file)

Analysis of OMICS data of control and HPFH samples

a) Analysis of RNA-seq data at T48; genes with differential ATAC peaks are indicated. **b)** Number of up- and downregulated genes in HPFH samples bound by KLF1, BCL11A, LRF or combinations thereof. **c)** Binding sites of KLF1, BCL11A, LRF or combinations thereof in human erythroid cells. **d)** Analysis of ATAC-seq data at T48. **e)** Differential ATAC peaks between HPFH individual 1 and 2 *versus* HPFH individual 3 and 4 at T48.

Supplementary Figures

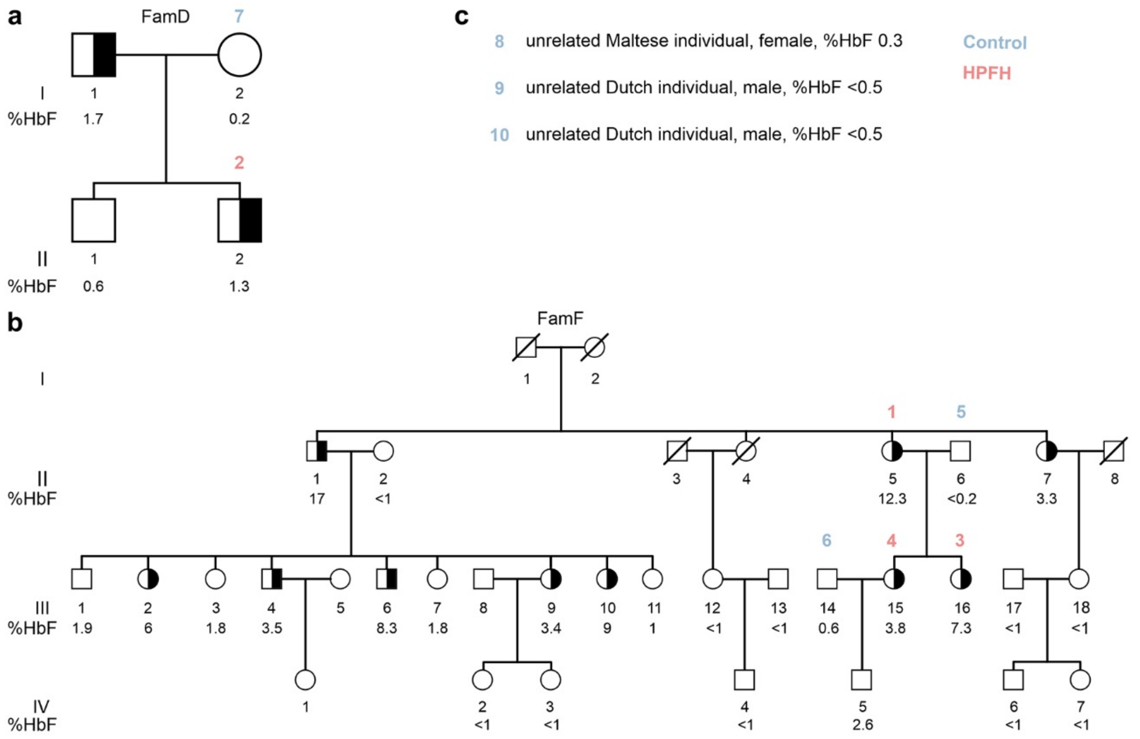

Supplementary Figure 1. Maltese HPFH pedigrees

(a) FamD **(b)** FamF **(c)** unrelated individuals used as controls in this study. Carriers of *KLF1* p.K288X are indicated by half-filled symbols. Individuals used in this study are indicated with light blue numbers (control individuals) and light red numbers (HPFH individuals). See also Table 1 and Suppl Table 1. FamF has been described previously in Borg *et al*, 2010 [8].

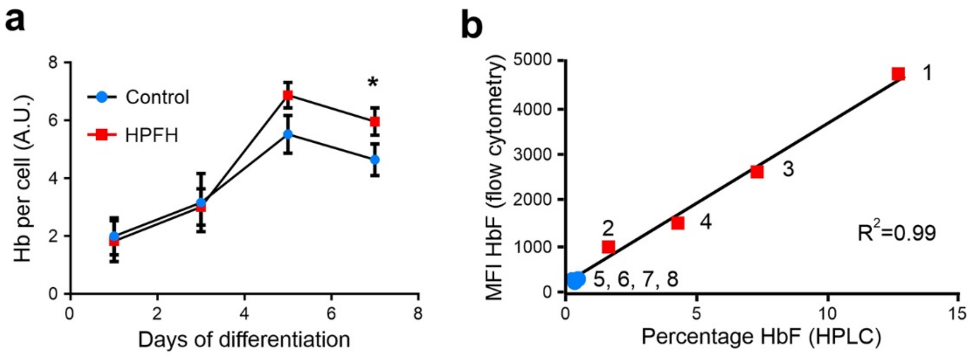

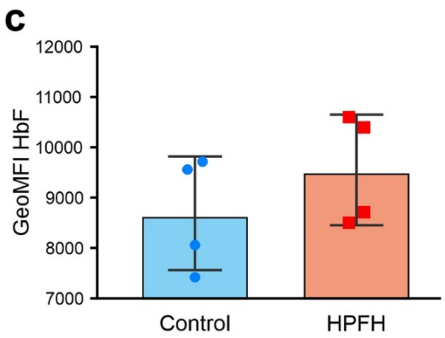

d

	Controls (n=4)		HPFH (n=4)		T-test
**T0**	% in gate	SD	% in gate	SD	p-value
CD71-CD235-	1.1	0.51	5.6	5.38	0.146
CD71+CD235-	42.4	13.79	44.8	9.80	0.790
CD71+CD235+	44.0	3.79	44.7	9.85	0.899
CD71-CD235+	13.2	11.44	5.0	1.91	0.207
**T48**	% in gate	SD	% in gate	SD	p-value
CD71-CD235-	2.2	1.55	3.5	1.68	0.355
CD71+CD235-	3.4	1.38	3.9	2.16	0.716
CD71+CD235+	73.5	4.13	77.7	3.95	0.233
CD71-CD235+	20.9	6.73	15.0	6.28	0.282

Supplementary Figure 2. Hemoglobin measurements on cultured cells and erythrocytes
(a) Hemoglobin assay shows comparable hemoglobinization between HPFH and control erythroid cells. **(b)** Line plot showing correlation between HbF levels measured in erythrocytes by HPLC (x-axis) and by flow cytometry Mean Fluorescence Intensity (MFI) measurements (y-axis). **(c)** Geometric Mean Fluorescent Intensity (GeoMFI of cells double-positive for HbA and HbF. **(d)** Flow cytometry of control and HPFH erythroid progenitor cultures grown under expansion conditions (T0) and after induction of differentiation (T48). * $p<0.05$, Students T-test. Error bars indicate SD.

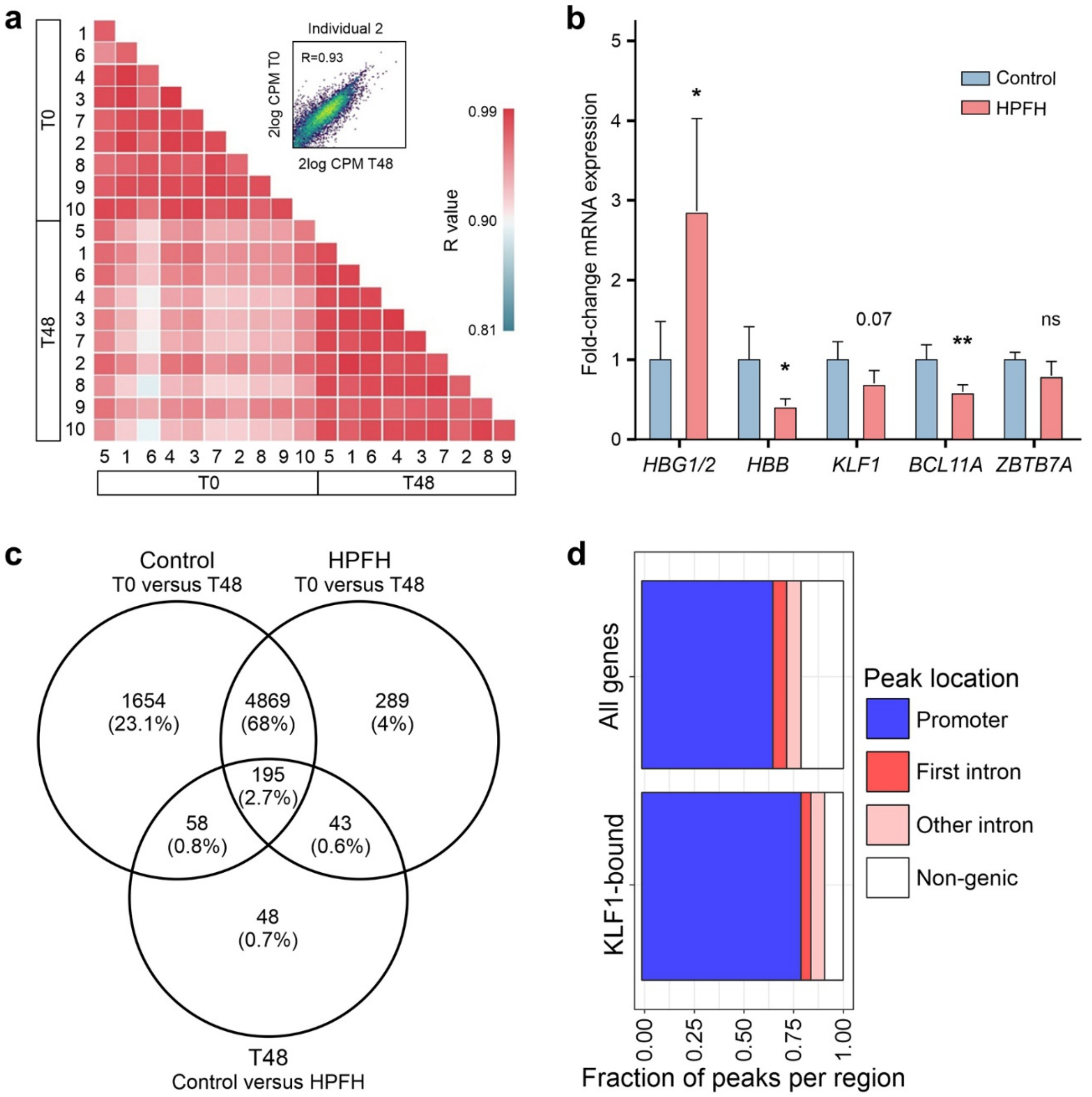

Supplementary Figure 3. Analysis of gene expression in cultured cells

(a) Correlation matrix between 2log-transformed count per million (CPM) values for RNA-seq samples. Label "T0" indicates expansion cultures and "T48" indicates cells grown for 48 hours in differentiation conditions as indicated in Supplementary Material and Methods. Insert shows 2log CPM expression plotted for a culture and differentiation sample from the same donor. The individuals from whom the samples were derived are indicated (Table 1). **(b)** RT-qPCR validation of expression of selected globins and regulators of hemoglobin switching. Expression levels in control samples were normalized to 1. **(c)** Venn diagram showing subdivision of differentially expressed genes between HPFH and control samples related to differentiation. **(d)** Location of KLF1 ChIP-seq peaks in differentially expressed genes between HPFH and control samples (170) compared to all annotated genes with a KLF1 peak (5298). * $p<0.05$; ** $p<0.01$, Students T-test. Error bars indicate SD.

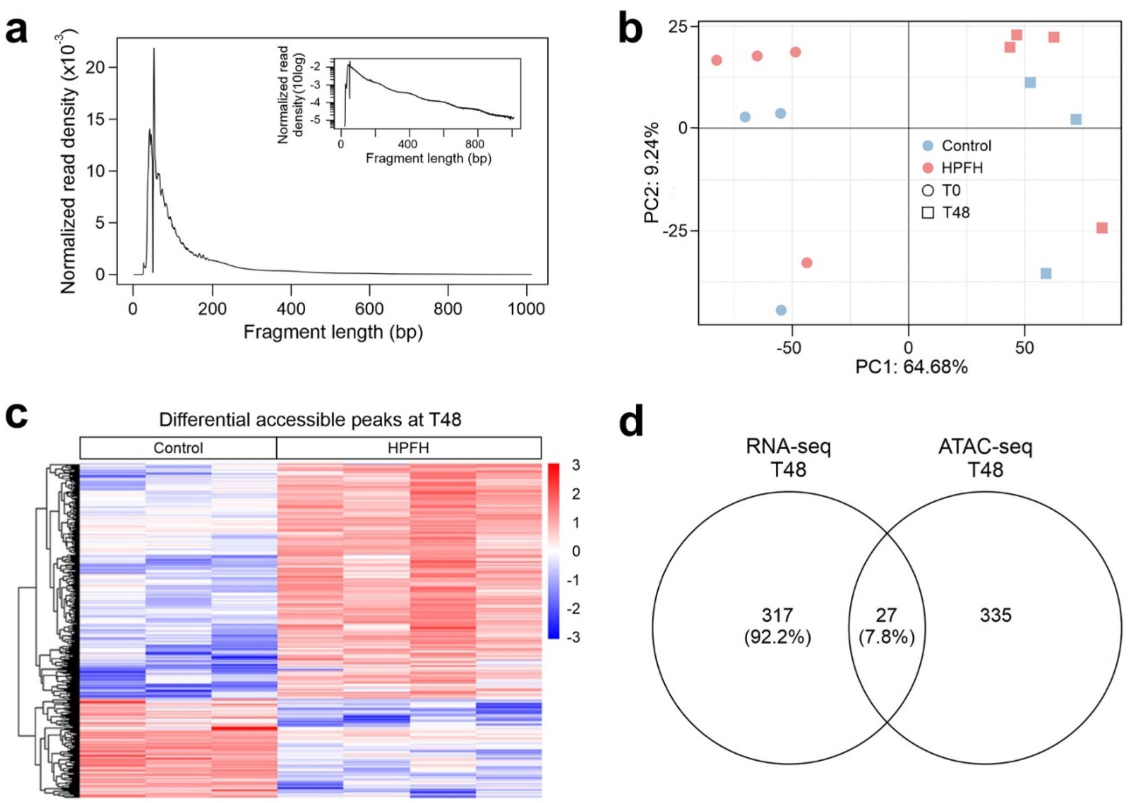

Supplementary Figure 4. Analysis of ATAC-seq data

(a) Density plots show insert size periodicity for a representative ATAC-seq sample. Insert shows log transformed insert size. Note the 200 bp periodicity. **(b)** Principal component (PC) analysis on 2000 most variable ATAC regions. **(c)** Heatmap showing z-transformed chromatin accessibility values for regions that are differentially accessible between HPFH and control cells at T48. **(d)** Venn diagram displaying the overlap between differential expression in RNA-seq and differential chromatin accessibility for uniquely mapped peaks in ATAC-seq. Of 559 differentially accessible ATAC peaks, 362 mapped to unique genes.

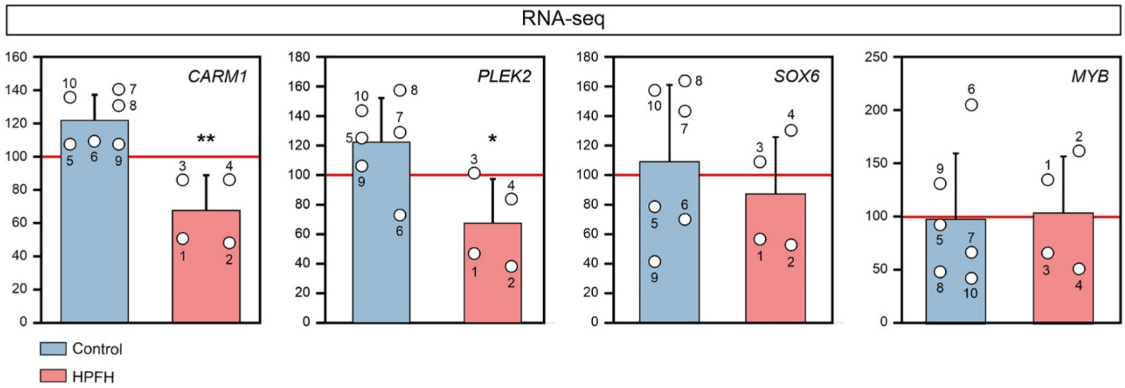

Supplementary Figure 5. Expression analysis of selected genes

Bar plots of mRNA expression derived from RNA-seq data. The individuals from whom the samples were derived are indicated (Table 1). The average expression level of all samples was normalized to 100 (red line). * p<0.05, ** p<0.01, Students T-test. Error bars indicate SD.
